# Microbiota in Breast Milk of Chinese Lactating Mothers

**DOI:** 10.1371/journal.pone.0160856

**Published:** 2016-08-16

**Authors:** Olga Sakwinska, Déborah Moine, Michèle Delley, Séverine Combremont, Enea Rezzonico, Patrick Descombes, Gerard Vinyes-Pares, Yumei Zhang, Peiyu Wang, Sagar K. Thakkar

**Affiliations:** 1 Nestlé Research Center, Lausanne, Switzerland; 2 Nestlé Institute of Health Sciences, Lausanne, Switzerland; 3 Nestlé Research Center Beijing, Beijing, People’s Republic of China; 4 Department of Nutrition & Food Hygiene, School of Public Health, Peking University, Beijing, People’s Republic of China; Universitat Ulm, GERMANY

## Abstract

The microbiota of breast milk from Chinese lactating mothers at different stages of lactation was examined in the framework of a Maternal Infant Nutrition Growth (MING) study investigating the dietary habits and breast milk composition in Chinese urban mothers. We used microbiota profiling based on the sequencing of fragments of 16S rRNA gene and specific qPCR for bifidobacteria, lactobacilli and total bacteria to study microbiota of the entire breast milk collected using standard protocol without aseptic cleansing (n = 60), and the microbiota of the milk collected aseptically (n = 30). We have also investigated the impact of the delivery mode and the stage of lactation on the microbiota composition. The microbiota of breast milk was dominated by streptococci and staphylococci for both collection protocols and, in the case of standard collection protocol, *Acinetobacter* sp. While the predominance of streptococci and staphylococci was consistently reported previously for other populations, the abundance of *Acinetobacter* sp. was reported only once before in a study where milk collection was done without aseptic cleansing of the breast and rejection of foremilk. Higher bacterial counts were found in the milk collected using standard protocol. Bifidobacteria and lactobacilli were present in few samples with low abundance. We observed no effect of the stage of lactation or the delivery mode on microbiota composition. Methodological and geographical differences likely explain the variability in microbiota composition reported to date.

## Introduction

A large number of studies have reported the presence of bacteria in breast milk of healthy lactating women. Early culture studies have showed the consistent presence of commensal bacteria in breast milk of healthy women, but bacteria were seen as a nuisance, complicating the storage and later use of expressed milk e.g. [1–5]. These early studies focused on the detection of potential pathogens and used culture methods that did not support the growth of organisms such as lactobacilli or bifidobacteria. The presence of coagulase-negative staphylococci (usually *S*. *epidermidis*), *S*. *aureus*, streptococci and *Acinetobacter* was consistently reported, while other taxa were found more sporadically (enterococci, *E*. *coli*, *Klebsiella*, *Moraxella*, *Pseudomonas*). Later studies applied methods allowing the growth of lactobacilli [6] and eventually also bifidobacteria [7–9], and these taxa were detected, but in low numbers (eg. bifidobacteria represented 1.7% and lactobacilli represented 0.4% of all isolated bacteria [8]).

Only recently has it has been increasingly considered that the presence of bacteria in various niches of the human body is not only a physiological norm, but can also be beneficial for the human host [10]. In parallel, the development of technology has permitted the investigation of bacteria in breast milk based on DNA signatures, initially by quantitative PCR targeting specific bacterial groups [11–17]. More recently, several studies have used microbiome profiling based on sequencing of fragments of 16S rRNA gene [8, 15, 16, 18, 19] and shotgun metagenomics consisting in sequencing of the entire bacterial DNA [20, 21]. The reported composition varied widely, suggesting that methodology as well as geographical or ethnical differences could play a role. Some studies observed composition similar to these obtained by culture based studies [16, 18, 21]; however, higher abundance of *Pseudomonas* than in culture based studies was reported [8, 16, 20, 21]. In one study the predominance of *Leuconostoc* and *Weisella* was observed [15]. The above studies were conducted in US or Western European populations. To our knowledge no studies investigating the microbiota of breast milk of lactating mothers were conducted in Asian populations. Most studies which used molecular techniques performed sterile collection, with rejection of the first few millilitres of breast milk. Only a few studies have investigated the microbiota of the entire breast milk collected without cleansing [19, 21] which the suckling infant actually ingests. Although, to our knowledge, no studies have compared the microbiota of the breast milk collected with aseptic cleansing with bacteria present in the entire milk, older studies using culture methods suggested that there were more bacteria in the foremilk [4].

The objective of this cross-sectional study was to investigate the microbiota of breast milk from Chinese lactating mothers during the different stages of lactation, using both aseptic collection protocol used by most studies to date, as well as standard collection protocol to target the breastfeeding-associated microbiota. Our study further substantiates the presence of bacteria in human milk, with a significantly higher number of bacteria identified in the “breastfeeding-associated microbiota” compared to milk obtained under aseptic conditions. We also confirmed the presence of the dominant species such as streptococci, staphylococci, as well as the low abundance of bifidobacteria and lactobacilli.

This study is part of the larger initiative Maternal Infant Nutrition Growth (MING) study.

## Methods

### Participants

This study was part of MING, a cross-sectional study designed to investigate the dietary and nutritional status of pregnant women, lactating mothers and young children aged from birth up to three years living in urban areas of China. In addition, the human milk composition of Chinese lactating mothers from 3 cities (Beijing, Guangzhou and Suzhou) was characterized in each city. The study was conducted between 2011 and 2012. Two hospitals with maternal and child care units were randomly selected. Furthermore, mothers at lactation period 0 to 240 days were randomly selected based on child registration information. Participants included in the period 0–5 days were recruited at the hospital whereas the other participants were requested by phone to join the study. If participation was dismissed a replacement was found. Response rate was 52%. In the present study of microbiota, only samples from lactating mothers from Beijing were used, and these were limited to three stages of lactation (0–4 days, 5–11 days and 1–2 months after birth). 60 participants chosen at random were included for microbiota analysis with standard collection protocol, and 30 with aseptic protocol, respectively (S1 Fig).

Women between 18–45 years of age who gave birth to a single, healthy, full-term infant and who were exclusively breastfeeding were included in the study. Exclusion criteria included gestational diabetes, hypertension, cardiac diseases, acute communicable diseases and postpartum depression. Lactating women who had nipple or lacteal gland diseases, who had been receiving hormonal therapy during the three months preceding recruitment, or who had insufficient skills to understand study questionnaires were also excluded. The study was conducted according to the guidelines in the Declaration of Helsinki. All of the procedures involving human subjects were approved by the Medical Ethics Research Board of Peking University (No.IRB00001052-11042). Written informed consent was obtained from all subjects participating in the study. The study was also registered in ClinicalTrials.gov with the number identifier NCT01971671.

### Data and sample collection

All participants responded to a general questionnaire including socio-economic and lifestyle aspects of the mother. The number of gestational weeks at delivery, delivery method, and the gender of the infant were recorded, the height and weight were measured. None of the participating mothers were treated with antibiotics. The samples were collected between 9:00 am and 11:00 am. The first set of thirty breast milk samples (10 samples/lactation stage collected from 30 mothers) were collected with an aseptic protocol. Sterile gloves were used, the first few drops (0.5–1 ml) were discarded, and the breast was thoroughly cleansed with chlorhexidine solution before manually collecting 3 ml of milk. The second set of sixty breast milk samples (20 samples/lactation stage collected from 60 mothers) were collected from a different group of mothers with standard protocol. Standard protocol did not include any particular cleaning procedure, and it was identical to the one used throughout MING study (S1 Fig, [22], Giuffrida et al in preparation, Affolter et al in preparation, Garcia Rodenas et al submitted). Single full breast milk was sampled with the use of an electric pump (Horigen HNR/X-2108ZB, Xinhe Electrical Apparatuses Co., Ltd), and after securing an aliquot of 10 ml (colostrum) or 40 ml (other lactation stages), the rest of the milk was returned to the mother for feeding to the infant. All the samples were frozen at -80°C before shipping to Lausanne, Switzerland for characterization, and they remained frozen until DNA extraction.

### DNA extraction

1 ml of milk was centrifuged for 20 minutes at 8000 g, and the pellets were dissolved in 500 μl of the supernatant. The first set of 30 samples was processed with the DNA Stool Mini kit (QIAGEN, Switzerland) according to the manufacturer’s instructions, with two modifications: the milk samples were heated at 95°C for 5 s and a bead-beating step with Fastprep (MP Biochemicals, USA) was added (30 s, speed 6 with 0.1 mm silica beads). The second set of 60 samples was processed with the Fast DNA SPIN Kit for soil (MO BIO, USA) according to the manufacturer’s instructions.

### Quantitative PCR

Quantitative PCR for total Bacteria, total Lactobacillus and total Bifidobacterium were performed as described in Table 1. All assays targeted variable regions of the 16S rRNA gene. The 25 μl PCR mixture contained TaqMan gene expression Master Mix (Applied Biosystems, Switzerland) or SensiMix Plus SYBR (Quantace, Switzerland), 2 μl of DNA extract, primers and Milli-Q water. The amplification reactions were performed in duplicate for each sample using the StepOnePlus Real-Time PCR System (Life Technology Europe, Switzerland). For each of the organisms standard curves were prepared by a tenfold dilutions of DNA from the reference strains within a range of 50 ng to 50 fg for Lactobacillus and Bifidobacterium and a range of 40 ng to 40 fg for total bacteria. The DNA concentration of the reference strains was measured by nanodrop ND-1000 (Nanodrop Technologies, USA). The results were expressed as genomic DNA copy numbers per sample volume (ml), based on the copy number present in the reference strains (Table 1).

**Table 1:** qPCR conditions.

Target	Reference strain	Primers 5’– 3’	Concentration of primers and probes (nM)	Detection limit (CFU/m)l	Cycling conditions	Reference
Total Bacteria	*Escherichia coli* K12	For: TCCTACGGGAGGCAGCAGT	300	5.5 x 10^2^	95°C 10’(95°C 15”/60°C 60”) 45x	[23]
		Rev: GGACTACCAGGGTATCTAATCCTGTT	300			
		Probe: CGTATTACCGCGGCTGCTGGCAC	175			
Total bifidobacteria Sample set collected with Standard protocol	*Bifidobacterium longum* NCC3001	For: CGATGCAACGCAAGAACC	500	1.4 x 10^3^	95°C 10’ (95°C 15”/60°C 60”) 50x	This study
		Rev: ATCTCACGACACGAGCTGAC	500			
		Probe: tt(C)gggg(S)ggttcaca	200			
Total bifidobacteria Sample set collected with Aspetic protocol	*Bifidobacterium lactis* NCC2818	g-Bifid-F: CTCCTGGAAACGGGTGG	250	1.4 x 10^2^	94°C 5’ (94°C 20”/55°C 20”/72°C 50”) 40x	[24]
		g-Bifid-R: GGTGTTCTTCCCGATATCTACA	250			
Total lactobacilli	*Lactobacillus johnsonii* NCC533	For: AGCAGTAGGGAATCTTCCA	500	2.4 x 10^2^	95°C 10’ (95°C 15”/58°C 20”/72°C 45”) 40x	[25]
		Rev: CACCGCTACACATGGAG	500			

### Microbiota profiling

For the 16S rRNA gene sequencing, the approach proposed by [26] was followed with some modifications. The V4 region was amplified by PCR (for 30μl reaction volume: 6μl of DNA, 1.2μl of each primer at 10 pM, 0.6μl of each dNTP at 2.5 mM, 6μl of Expand High Fidelity PLUS Reaction Buffer, 0.3μl of Expand High Fidelity PLUS Enzyme Blend 5U/μl (Roche Applied Science, Switzerland). Amplification conditions were the following: 94°C for 2 min followed by 30 cycles of 94°C for 30 seconds, 50°C for 30 seconds and 72°C for 30 seconds followed by 7 minutes at 72°C and finally held at 4°C). The primers mapped to the conserved regions flanking V4 and produced an amplicon of 425 bp. In addition, they carried a specific barcode to multiplex the samples for sequencing and the Illumina sequencing adapter. The primers were identical to these used in [26]. After quantification and quality check by capillary electrophoresis (LabChip GX, Perkin Elmer, USA), all the amplicons were pooled in equimolar amount and purified. In cases where the DNA concentration was below the detection limit, all available sample volume was used for pooling. Sequencing was performed on the MiSeq instrument V2.1 (with Hard Coded matrix for data acquisition) paired-end 250 bp (MS-102-2003). Demultiplexing was performed by the MiSeq software (V2.1). Raw sequence data were deposited in the GenBank Short Read Archive (Accession number: SRP077712).

### Processing of the sequencing data

Data processing was done with mothur software v.1.32.1 [27]. Paired-end sequences were joined as described [28]. The sequences were aligned to Silva reference alignment and trimmed so that complete overlap was achieved. A preclustering step with 2%-linkage algorithm was performed [29]. Chimeras were removed using uchime algorithm using de novo option [30]. The sequences were classified with RDP classifier (version 9) using 0.8 confidence level. The sequences were clustered into OTUs using 0.03 similarity cut-off value. The distances among sequences were calculated as pairwise uncorrected distance, where gaps were considered a single position, and clustering using average neighbor method. Before calculating beta diversity measures and statistical analyses, the number of sequences per sample was made uniform by random subsampling of 2226 reads from each sample. This eliminated samples that had fewer than 2226 sequencing reads: 8 out of 30 samples from the first data set, and 2 out of 60 samples from the second data set.

## Results

### Participants’ characteristics

S1 Fig displays the recruitment flowchart from eligibility to sample analysis. Demographics and anthropometry of the participants are described in Table 2. Although there was no specific selection to exclude smokers, there were only few smokers in the investigated population and all mothers in this subset were non-smokers.

**Table 2:** Maternal and infant characteristics.

Variable	Aseptic protocol (n = 30)	Standard protocol (n = 60)
**Mother**		
Age (years), Mean (SD)	28.1 (3.6)	28.2 (3.7)
Height (cm), Mean (SD)	163 (5.1)	162 (5.4)
Weight (kg), Mean (SD)	63.4 (7.8)	65.4 (9.5)
BMI (kg/m2)	23.9 (3.3)	25.1 (3.5)
Caesarean delivery, N (%)	13 (43)	33 (55)
**Infant**		
Males, N (%)	13 (48)	21 (37)
Gestational age at birth (weeks), Mean (SD)	39.5 (1.1)	39.2 (1.2)

### Quantification of bacteria by qPCR

**Fig 1:**
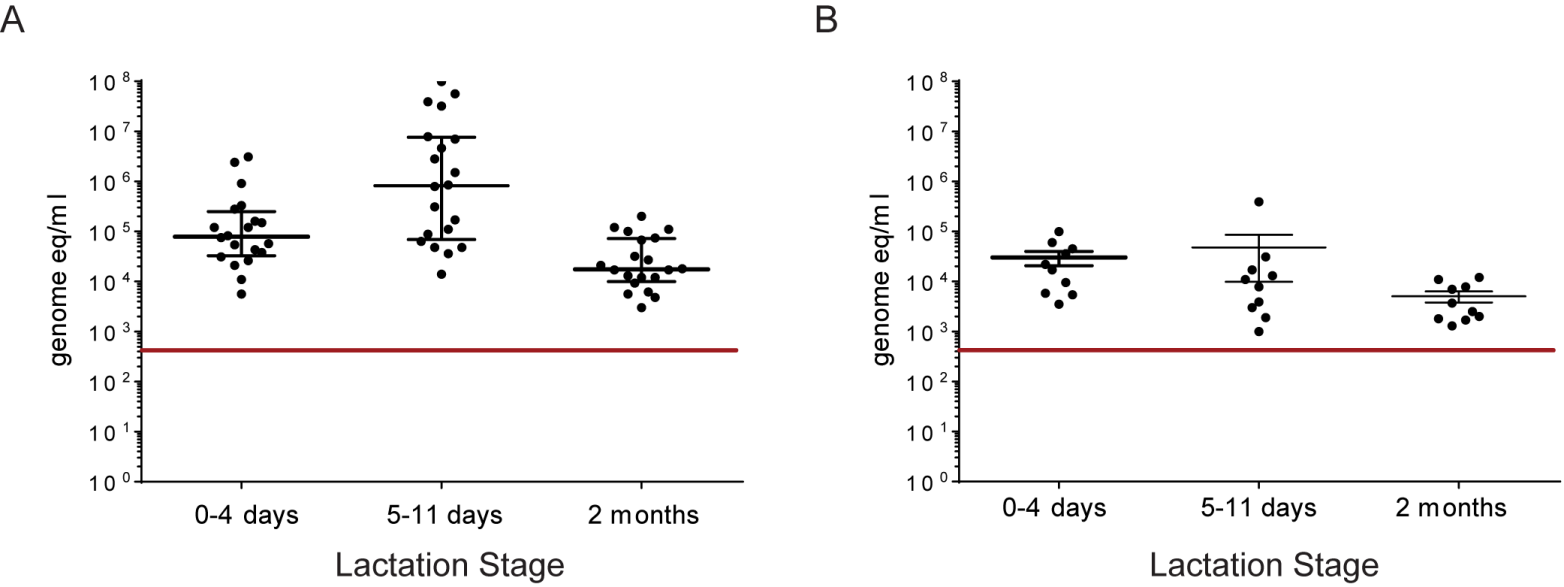
Quantification of bacteria by qPCR. Total bacteria counts measured by qPCR in samples collected with standard (A) and aseptic protocol (B) at three lactation stages. Detection limits are shown by red line.

Total bacterial load measured by qPCR (Fig 1) was higher in breast milk collected with standard protocol (median 7.5 x 10^4^ counts/ml) than in samples taken with aseptic precautions (median 7.8 x 10^3^ counts/ml). This difference was significant for samples taken at 5–11 days (Mann-Whitney test, p<0.0001), but not at 0–4 days (Mann-Whitney test, ns) or at 2 months (Mann-Whitney test, ns). Bifidobacteria and lactobacilli were found in only few samples (Table 3). It has to be noted that a different method of detection of bifidobacteria was used for the second set of samples (taken without aseptic precautions), resulting in a lower detection limit.

**Table 3:** Detection of lactobacilli and bifidobacteria in breast milk samples. Number of positive samples is shown.

Condition	n	Bifidobacteria	Lactobacilli
**Aseptic Protocol**			
0–4 days	10	2	2
5–11 days	10	0	1
2 months	10	0	0
**Standard protocol**			
0–4 days	20	2	0
5–11 days	20	0	0
2 months	20	1	2

### Microbiota profiling by sequencing of 16S rRNA gene amplicons

The quantity of bacteria in the set of breast milk samples collected with aseptic protocol was mostly below the limit usually considered sufficient for microbiota profiling based on 16S rRNA gene sequencing (10^6^ genome copies/ml) [31]. The majority of samples (23) gave no quantifiable PCR product. Out of 30 samples, only one yielded adequate PCR amplicon and six further samples gave weak products. The samples collected with standard protocol contained more bacterial DNA, and out of 60 samples 17 yielded adequate PCR product, 10 samples yielded weak PCR product, and 33 gave no quantifiable PCR product. Despite the fact that many samples did not reach standard quality requirements for sequencing, all samples were sequenced. The samples which gave good amplification products were compared to those with week amplification products, and no differences were seen (S2 Fig).

**Fig 2:**
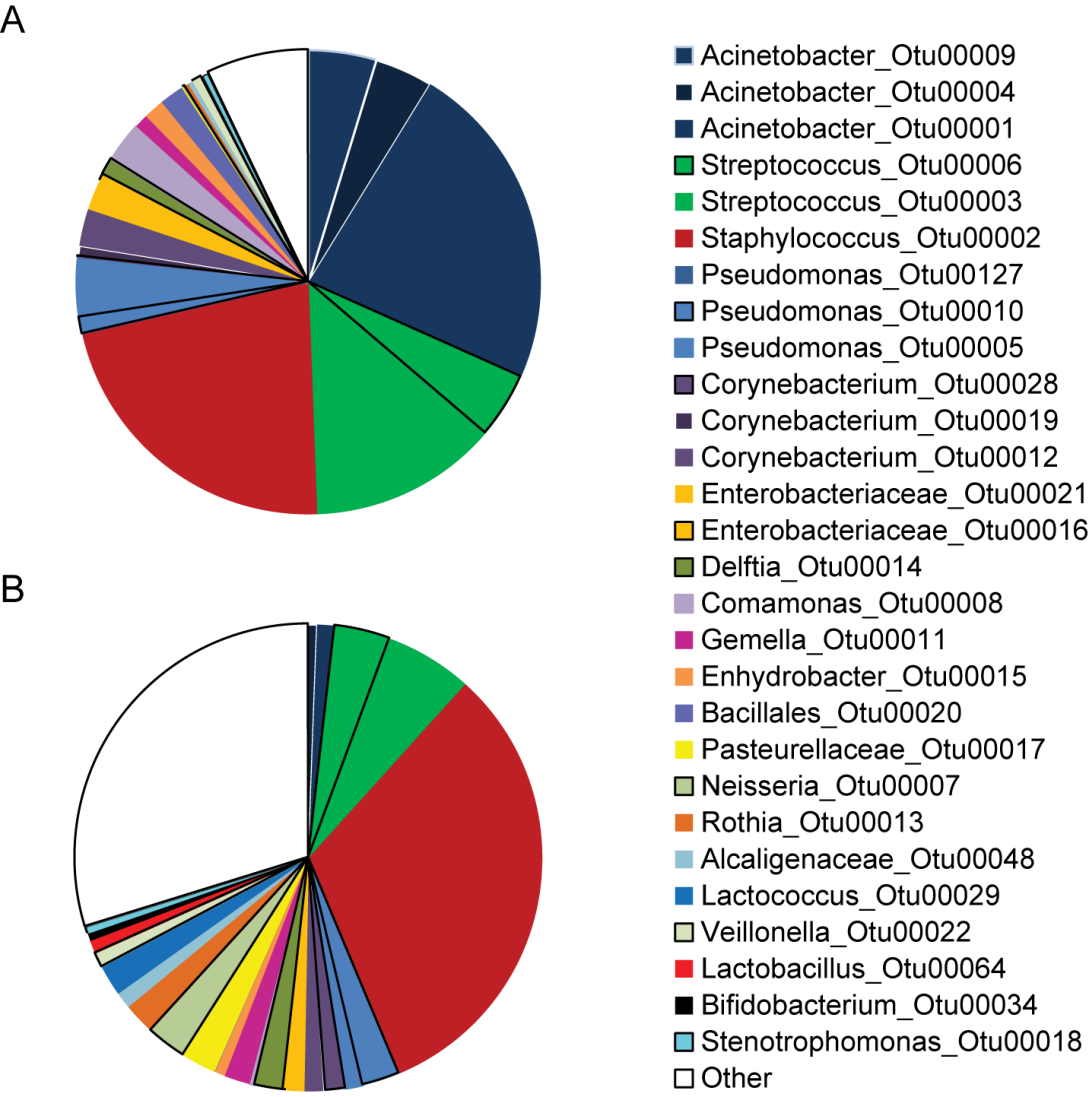
Average microbiota profiles. Microbiota composition in samples collected with standard (A) and aseptic protocol (B). Average abundances of OTUs are shown. Sequences belonging to OTUs which contained smaller number of sequences were pooled and are labeled “Other”.

Staphylococci and streptococci were equally abundant in both sets of samples, on average 42% and 40% of total microbiota in samples collected using the aseptic and the standard protocol respectively (Fig 2). High abundance of *Acinetobacter* sp. (average 32%) was seen in samples collected with standard protocol, but not in samples collected with strict aseptic protocol (average 1.8%).

**Fig 3:**
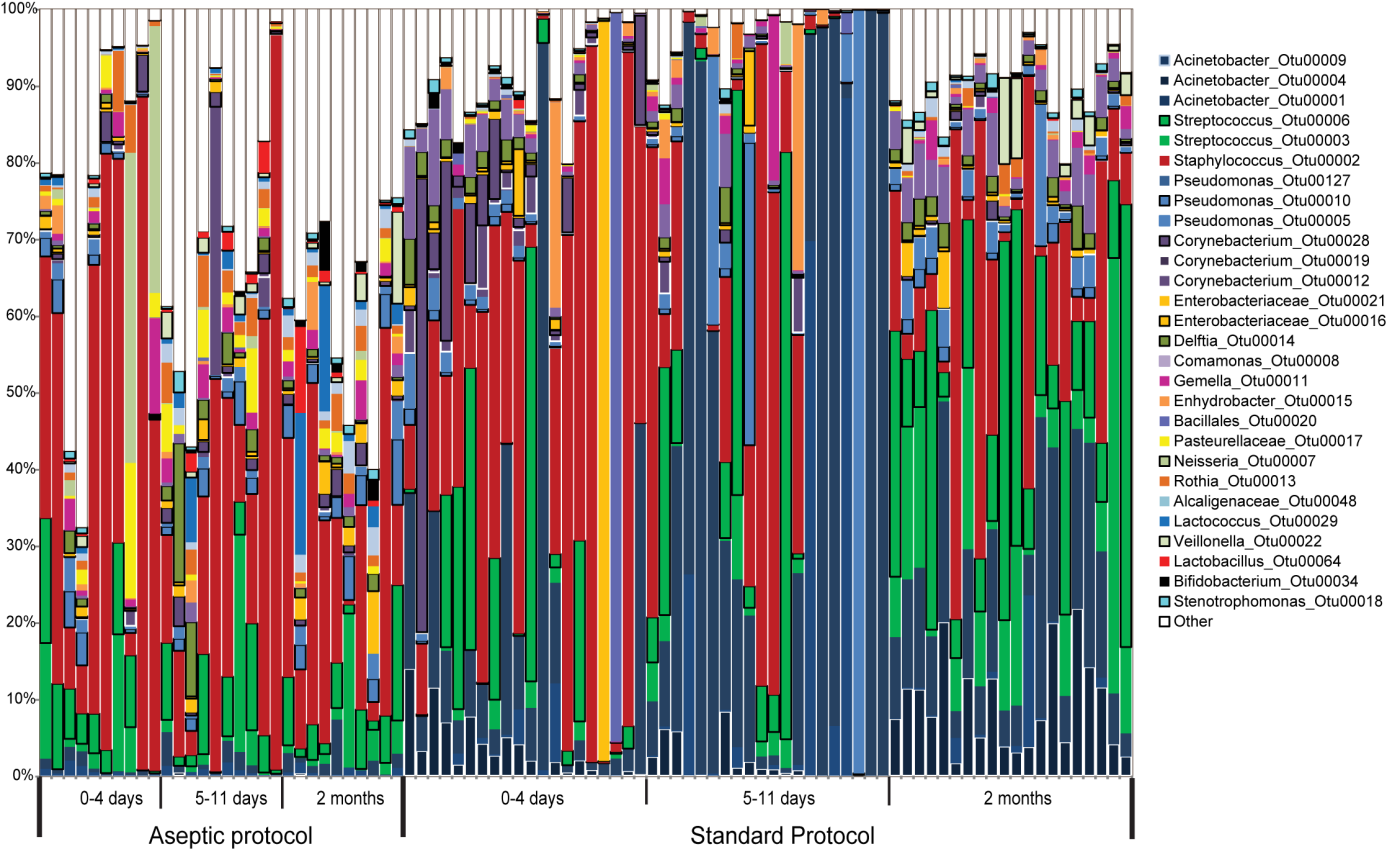
Microbiota profiles of individual subjects. The composition of breast milk microbiota collected with aseptic and standard protocol at different stages of lactation. The 28 most abundant OTUs are shown. Sequences belonging to OTUs which contained smaller number of sequences were pooled and are labeled “Other”.

High intra-individual variability in microbiota composition was observed (Fig 3). Low proportion of lactobacilli in the sample set collected with aseptic protocol (0.9%) and standard protocol (0.03%) and bifidobacteria (0.5% and 0.15%, respectively) was consistent with qPCR results (Table 3).

**Fig 4:**
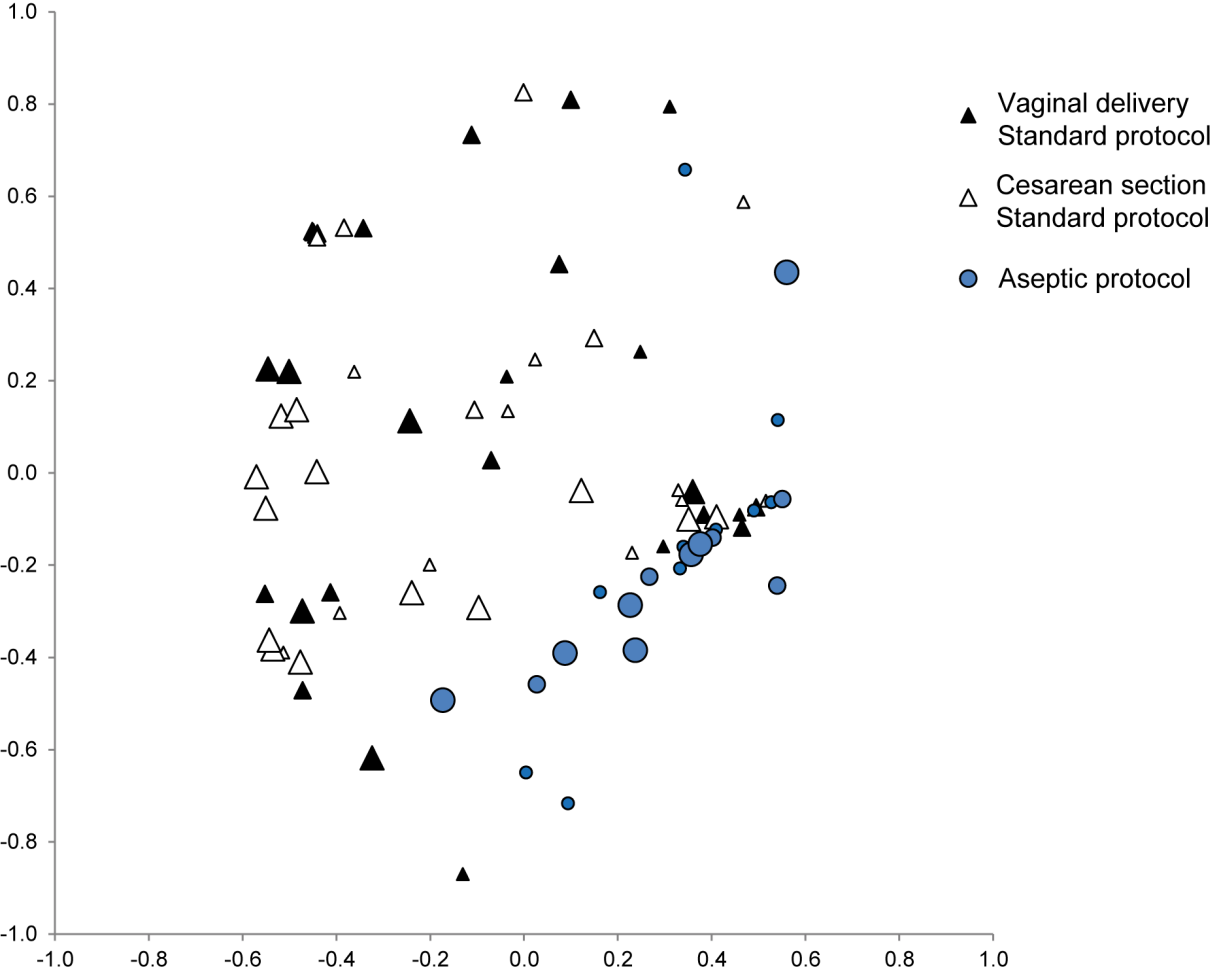
Multivariate analysis. Non-metric multidimensional scaling (NMDS) ordination plot, showing axis 1 and 2. The input data for ordination plots were Yue & Clayton measure of dissimilarity, based on microbiota composition at operational taxonomic unit level. The size of the points represents the time of lactation, the smallest points corresponding to days 0–4 days, the medium to days 5–11, and the largest to month 2.

Multivariate NMDS analysis showed significant differences between the samples collected with standard and aseptic protocols (AMOVA, p<0.001), but no differences due to the stage of lactation or the delivery mode (AMOVA, ns), (Fig 4).

## Discussion

### Microbiota profiles

To our knowledge, this is the first report on microbiota composition in a large cohort of Chinese mothers. The microbiota of breast milk from Chinese mothers was dominated by streptococci, staphylococcci and, in the case of standard collection protocol, *Acinetobacter* sp. The composition of microbiota in breast milk reported previously varied considerably, but the high abundance of staphylococcci and streptococci was perhaps the most consistent feature in the majority of studies which used microbiota profiling (Table 4).

**Table 4:** Studies with characterization of bacteria present in breast milk by microbiota profiling.

Predominant taxa	Characterization method	Abundance of bifidobacteria and lactobacilli	N	Population	Collection protocol	Reference
*Staphylococcus sp*., *Streptococcus sp*.	Pyrosequencing	“very few sequences”	16	US	Cleansing with iodine	[18]
					Entire breast	
*Leuconostoc sp*. *Weisella sp*.	Pyrosequencing	No *Bifidobacterium or Lactobacillus reported*	18	Finnish	Cleansing with iodine	[15]
					Rejection of first drops	
*Staphylococcus sp*., *Pseudomonas sp*., *Streptococcus sp*.,	Pyrosequencing	*Bifidobacterium* 1.3% *Lactobacillus* absent	7	Swiss	Cleansing with aseptic soap	[8]
					Rejection of foremilk	
*Staphylococcaceae*, *Streptococcaceae*, *Pseudomonadaceae*	Pyrosequencing	No *Bifidobacterium or Lactobacillus reported*	10	Spanish	Cleansing with chlorhexidine	[36]
					Rejection of first drops	
*Acinetobacter sp*., *Staphylococcus sp*.	Ion Torrent	*Bifidobacterium* 2% *Lactobacillus* 1.6%	8	US	Cleansing with saline	[19]
					First 10 to 15 ml taken	
*Staphylococcus sp*., Unclassified genus of *Enterobacteriaceae*, *Pseudomonas sp*., *Streptococcus sp*.,	Illumina	*Bifidobacterium* 0.8% *Lactobacillus* 3%	39	USA Caucasian	Cleansing with saline	[37]
					First 5 to 15 ml taken	
*Staphylococcus sp*., *Pseudomonas sp*.,	Metagenomics	No *Bifidobacterium Lactobacillus* 0.2%	10	US	None	[21]
*Staphylococcus sp*., *Pseudomonas sp*.,	Metagenomics	*Bifidobacterium* 0.1% *Lactobacillus* 0.6%	10	Spanish	Cleansing with chlorhexidine	[20]
					Rejection of first drops	

The predominance of staphylococci and in most studies also streptococci was universally observed in early studies based on culture methods [1–5]. These early studies treated the bacterial presence as contamination so naturally focused on the detection of potential pathogens. However, the dominance of staphylococci and streptococci was confirmed more recently when a more diverse range of culture media to cultivate bacteria was used [6, 8, 9].

Our study is consistent with this pattern, suggesting that the microbiota of breast milk from Chinese lactating mothers is similar to that seen at other geographic locations. However, further studies in this and other Asian populations are needed before more definitive conclusions can be drawn.

Only a few studies have investigated the microbiota of the entire breast milk collected without aseptic cleansing, which we propose to call “breastfeeding-associated microbiota” [19]. We considered that breastfeeding-associated microbiota is relevant, as it represents the bacteria ingested by the suckling infant. It is of note that in a study where high abundance of *Acinetobacter* sp. was reported by [19], milk collection was done without aseptic cleansing of the breast and rejection of foremilk, similarly to the approach we used in the present study (Table 4). Although in our study the experimental methods, such as the extraction protocol, differed between the two sets of samples, we find it unlikely that this was a reason for the predominance of *Acinetobacter* sp. in samples collected with standard protocol. It remains to be determined whether the high abundance of *Acinetobacter* sp. is a specific feature of breastfeeding-associated microbiota.

While PCR amplification of samples proved to be difficult, most likely due to low numbers of bacteria, we feel confident that our results reflect the actual composition of microbiota. Indeed we obtained relatively similar results when considering exclusively the samples with good amplification, as indicated by lack of clustering seen in multivariate NMDS analysis. The bacteria were more abundant in the milk collected with standard protocol without rejection of the first drops, which is in agreement with an early study based on bacterial culture [4] where the median number of bacteria decreased ten times, from approximately 10^3^ to 10^2^ CFU per ml, when the first milliliters of breast milk were rejected. In other studies the reported total quantity of live bacteria in breast milk varied, but most studies reported median numbers of 10^2^ to 10^3^, and a range from 10^1^ to 10^7^ CFU per ml of breast milk [1–3, 5–8, 32–35]. One early study suggested that using breast milk pump leads to increased bacterial counts as compared to manual milk expression [1].

In most of the studies, these numbers represent bacteria present inside the breast because the samples were taken with aseptic precautions and elimination of the first few microliters to milliliters of milk. As mentioned above, the numbers present in the entire breast milk appeared higher, but only one early study examined this question directly [4].

Of note, a recent study examined the bacteria present in breast milk tissue of non-lactating women [19]. Low (up to 10^3^CFU/g tissue) numbers of skin commensals were found, suggesting that the presence of live bacteria inside the breast is not limited to the period of breastfeeding.

### Abundance of lactobacilli and bifidobacteria

The abundances of bifidobacteria and lactobacilli in microbiota profiles found in our study are in agreement with previously published data (Table 4). Previous studies of breast milk using qPCR detection techniques yielded considerably variable results (summarized in Table 5). In particular, a previous study [15] reported high abundance of bifidobacteria; in addition, bifidobacteria constituted a considerable proportion of the entire bacterial population (Tables 4 and 5). However, the high abundance of bifidobacteria and lactobacilli measured by qPCR was not seen in microbiota profiles [15]. Studies which employed culture techniques rarely reported quantitative results and if reported, low abundance of bifidobacteria and lactobacilli were seen: [8]—1.7% of bifidobacteria and 2.1% lactobacilli; [6]—no culture of bifidobacteria performed and 1.8% of lactobacilli; [7]—0.2% of bifidobacteria and 0.4% of lactobacilli. One study where no sterile cleansing of the breast was performed [9], found higher proportions of lactobacilli and bifidobacteria (5% each of all isolates). A recent study using culture methods [35] found that 40% of samples positive for lactobacilli and 11% were positive for bifidobacteria while the average counts of positive samples were 10 cells per ml for both taxa.

**Table 5:** Studies with characterization of bacteria present in breast milk by quantitative PCR.

Abundance of Lactobacilli	% bacterial population^1^	Abundance of Bifidobacteria	% bacterial population^1^	N	Population	Collection protocol	Reference
Not tested	Not tested	3.9E+2	Not tested	20	Finnish	Not specified	[13]
Not tested	Not tested	1.4E+3	Not tested	61	Finnish	Samples collected after infant has suckled on the breast	[12]
Not tested	Not tested	3.4E+3	2.8%	23	Spanish	Cleansing with chlorhexidine	[14]
						Rejection of first drops	
3.7E+3	<0.01%	3.6E+3	<0.01%	50	Spanish	Cleansing with chlorhexidine	[11]
						Rejection of first drops	
9.6E+5	197%	3.3E+5	68%	56	Finnish	Cleansing with iodine	[15]*
						Rejection of first drops	
2.7E+4	72%	2.7E+4	54%	18	Finnish	Cleansing with iodine	[38]*
						Rejection of first drops	
2.1E+4	40%	1.7+2	0.3%	32	Spanish	Cleansing with chlorhexidine	[17]
1.8E+4	44%	1.6+2	0.4%	10	Spanish	Cleansing with chlorhexidine	[36]
						Rejection of first drops	

^1^ Based on division of the counts of bifidobacteria and lactobacilli by total bacterial counts

*Different subsets of the same study, NCT00167700

Methodological and geographical differences may explain some of this variability. The low number of bacteria found in breastmilk samples very likely contributes to the variability. The qPCR methods used were initially developed for stool samples, where bacterial loads are five to seven orders of magnitude higher than in breast milk. In the culture based studies, lack of standardization of the details of sample handling such as time to plating and oxygen exposure likely contributed to the variability of results.

### The impact of the stage of lactation and the delivery mode

We found no effect of the delivery mode or the stage of lactation on microbiota composition, although this last conclusion is limited to the first 2 months of lactation. Three previous studies evaluated the impact of the stage of lactation on the bacterial counts of total bacteria, lactobacilli or bifidobacteria by qPCR [15, 17, 38] and one of them [17] found that the counts of bifidobacteria increased with the time of lactation, while the remaining studies found no differences. A recent study [34] using culture methods also showed the increase in the counts of bifidobacteria with the time of lactation. The impact of the stage of lactation was also investigated by microbiota profiling [15, 17, 38], but statistical tests to investigate the differences were not reported.

Concerning the impact of the delivery mode, one study [17] found slightly higher counts of total bacteria in colostrum and transition milk of women who delivered by caesarean section, compared to those who delivered vaginally, while this difference was not seen in mature milk. The counts of lactobacilli and bifidobacteria did not differ between the three stages of lactation. It was reported [15] that, apart from slightly higher counts of bifidobacteria in colostrum of women who delivered vaginally (p = 0.05) compared to those who delivered by caesarean section, no differences were seen in the bacterial counts of total bacteria and lactobacilli at three stages of lactation, and in bifidobacteria counts in transition and mature milk. [36] found no differences in the counts of total bacteria, lactobacilli or bifidobacteria between the two delivery modes. Microbiota profiles were described in [15, 36], but no statistical tests to assess the differences between the delivery modes were presented. The differences between the delivery modes were again assessed in a recent study [37] and no significant differences in microbiota composition were found.

Overall, no consistent patterns were found regarding the impact of the delivery mode and the time of lactation on the microbiota of breast milk.

In conclusion our study further substantiates the presence of bacteria in human milk, with a significantly higher number of bacteria identified in the “breastfeeding-associated microbiota” compared to milk obtained under aseptic conditions. We also confirmed the presence of the dominant species such as streptococci, staphylococci, as well as the low abundance of bifidobacteria and lactobacilli.

## Supporting Information

S1 Fig The recruitment flowchart from eligibility to sample collection. (PDF)

S2 Fig Multivariate analysis. Non-metric multidimensional scaling (NMDS) ordination plot, showing axis 1 and 2. The input data for ordination plots were Yue & Clayton measure of dissimilarity, based on microbiota composition at operational taxonomic unit level. The difference on overall microbiota composition between samples that gave good PCR product (PCR pos), and those that gave weak PCR product (PCR neg) was not significant (AMOVA).
(PDF)
