# Supplementary figures and images for: Microbiota in Breast Milk of Chinese Lactating Mothers
Source: PLoS One. 2016 Aug 16;11(8):e0160856. doi: 10.1371/journal.pone.0160856 (PMC4987007; doi:10.1371/journal.pone.0160856)

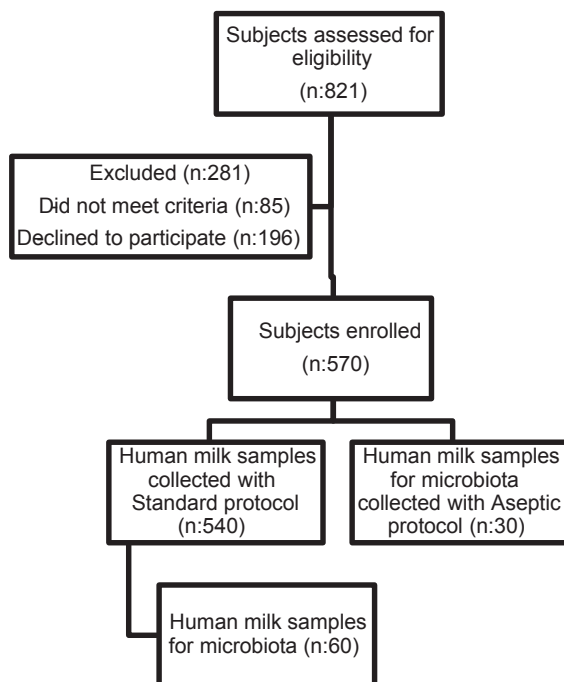

Supplement: S1 Fig — (PDF) [file pone.0160856.s001.pdf]

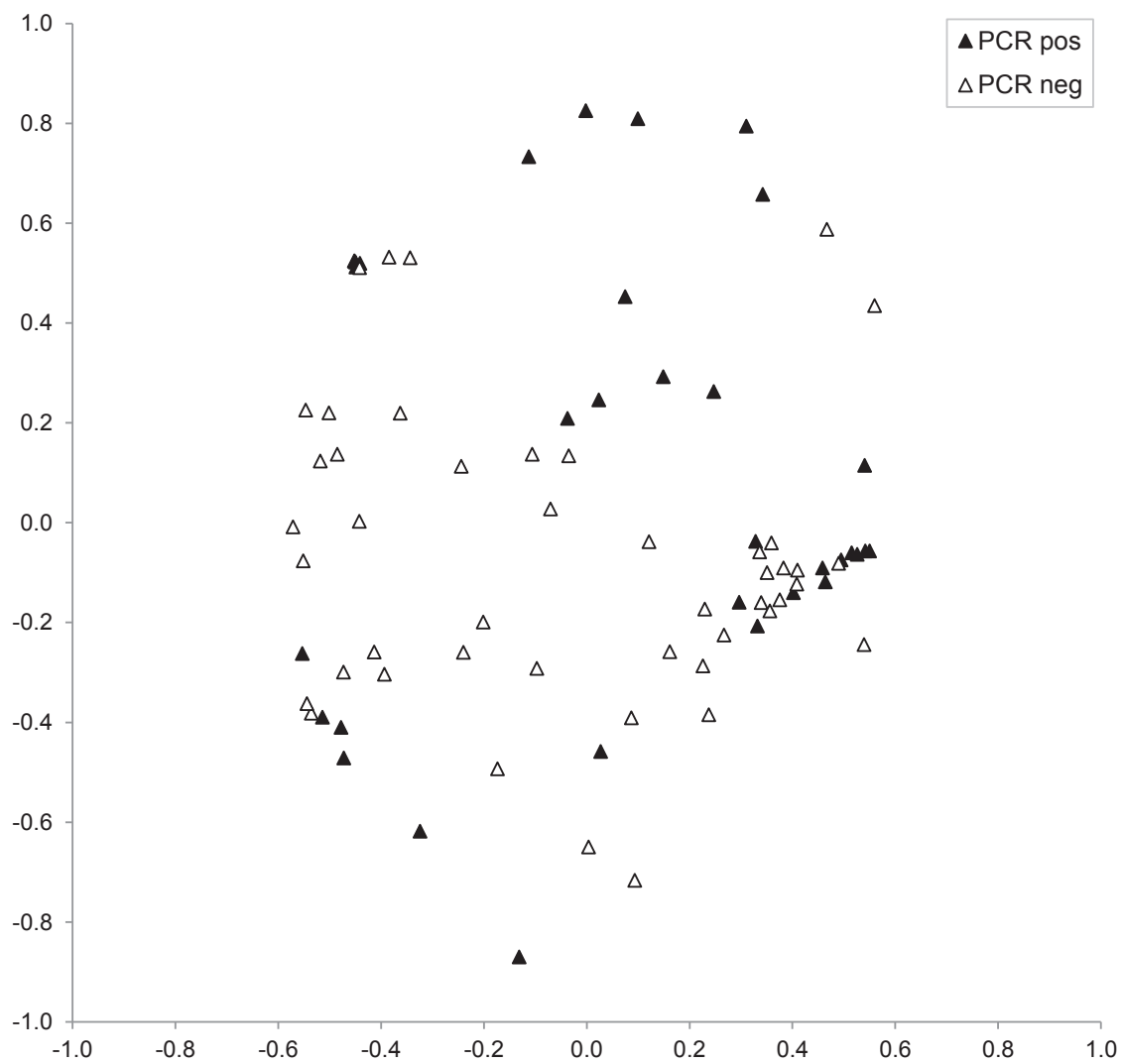

Supplement: S2 Fig — Non-metric multidimensional scaling (NMDS) ordination plot, showing axis 1 and 2. The input data for ordination plots were Yue & Clayton measure of dissimilarity, based on microbiota composition at operational taxonomic unit level. The difference on overall microbiota composition between samples that gave good PCR product (PCR pos), and those that gave weak PCR product (PCR neg) was not significant (AMOVA). (PDF) [file pone.0160856.s002.pdf]
